# Rewiring of Microbiota Networks in Erosive Inflammation of the Stomach and Small Bowel

**DOI:** 10.3389/fbioe.2020.00299

**Published:** 2020-05-13

**Authors:** Xiao-Yu Chen, Hui-Ning Fan, Huang-Kai Zhang, Huang-Wen Qin, Li Shen, Xiang-Tian Yu, Jing Zhang, Jin-Shui Zhu

**Affiliations:** ^1^Department of Gastroenterology, Shanghai Jiao Tong University Affiliated Sixth People’s Hospital, Shanghai, China; ^2^Aginome-XMU Joint Laboratory, Xiamen University, Xiamen, China; ^3^Clinical Research Center, Shanghai Jiao Tong University Affiliated Sixth People’s Hospital, Shanghai, China

**Keywords:** magnetically guided capsule endoscopy, metagenomics, edge-network analysis, chronic gastritis, small bowel erosion

## Abstract

The development of non-invasive, inexpensive, and effective early diagnosis tests for gastric and small-bowel lesions is an urgent requirement. The introduction of magnetically guided capsule endoscopy (MGCE) has aided examination of the small bowel for diagnoses. However, the distribution of the fecal microbiome in abnormal erosions of the stomach and small bowel remains unclear. Herein, alternations in the fecal microbiome in three groups [normal, small-bowel inflammation, and chronic gastritis (CG)] were analyzed by metagenomics and our well-developed method [individual-specific edge-network analysis (iENA)]. In addition to the dominant microbiota identified by the conventional differential analysis, iENA could recognize novel network biomarkers of microbiome communities, such as the genus *Bacteroide* in CG and small-bowel inflammation. Combined with differential network analysis, the network-hub microbiota within rewired microbiota networks revealed high-ranked iENA microbiota markers, which were disease specific and had particular pathogenic functions. Our findings illuminate the components of the fecal microbiome and the importance of specific bacteria in CG and small-bowel erosions, and could be employed to develop preventive and non-invasive therapeutic strategies.

## Introduction

Choung and colleagues reported that in the United States, 338 out of 31,255 adult residents were diagnosed with diseases of the digestive system (Choung et al., 2017). In particular, chronic gastritis (CG) and small-bowel erosion account for >60% of cases diagnosed by magnetically guided capsule endoscopy (MGCE) in China (Ding et al., 2019) and South Korea (Lim et al., 2015). Introduction of MGCE has expanded clinical diagnosis of gastrointestinal diseases, including CG and small-bowel erosion (Aktas and Mensink, 2012). However, the unbiopsied state, high cost, and time consumption of MGCE limit its application in diagnoses (Costamagna et al., 2002; Niv and Niv, 2005). Development of non-invasive, effective detection in small-bowel lesions is needed urgently.

Metagenomics using next-generation sequencing (mNGS) is becoming a promising approach for identifying the microbiome community in human diseases (Wilson et al., 2019). Some studies have shown an increased ratio of Basidiomycota:Ascomycota, increased proportion of *Candida albicans*, and a decreased abundance of *Saccharomyces cerevisiae* in inflammatory bowel disease (IBD) (Sokol et al., 2017). Significant differences in levels of bacterial genera have been used to detect atrophic gastritis/intestinal metaplasia, and gastrointestinal tumors (Coker et al., 2017; Zhang et al., 2019). mNGS can also be used to distinguish the functions of the gut microbiome in IBD and irritable bowel syndrome (IBS) (Vich Vila et al., 2018). Small-bowel microbiota not only regulate assimilation of the adaptive responses to lipids in germ-free mice (Martinez-Guryn et al., 2018) but also act by assessing the small-bowel damage induced by non-steroidal anti-inflammatory drugs (Otani et al., 2017). However, use of the fecal microbiome for identification of gastric and small-bowel abnormalities has not been done.

An accurate clinical diagnosis can enable monitoring, quantification, and progression of a disease (Zeng et al., 2014; Yu et al., 2015), and can be realized using sample-specific biomarkers (Zeng et al., 2016). Previously, we proposed an individual-specific edge-network analysis (iENA) to detect the early warning signals or pre-disease state before disease onset (Yu et al., 2017). Also, we carried out proof-of-concept research on the rewiring community of intestinal ecosystems by an adjusted iENA method on the basis of 16S rRNA data (Wang et al., 2018; Yu et al., 2019).

Here, we used the method of computational systems biology that we had developed to analyze the dominant microbiota and network on the basis of fecal metagenomics data. We identified specific bacteria that had key roles in the clinical classification of erosive lesions of the small bowel and CG that might offer prevention and non-invasive treatment strategies.

## Materials and Methods

### Ethical Approval of the Study Protocol

The study protocol was approved by the Ethics Committee of Shanghai Sixth People’s Hospital, which is affiliated with Shanghai Jiao Tong University (Shanghai, China). Written informed consent was obtained from all individuals. Personal data were anonymized and omitted.

### Study Enrollment

The study ran at Shanghai Sixth People’s Hospital from May 1, 2017 to September 1, 2018. The individuals who agreed to complete examinations of MGCE (Ankon Medical Technologies, Shanghai, China) and mNGS examination of their stools (*n* = 15) were recruited. The procedures of enrollment, fecal mNGS, and MGCE classification were completed independently by different investigators who were blinded to the results of each other’s examinations. Fisher’s exact test was used for evaluation of statistical difference in comparisons between three groups. *p* < 0.05 was considered statistically significant.

### MGCE and Stool Collection

At least three stool samples were collected from each eligible individual and stored at −80°C. All patients underwent intestinal preparation with an electrolyte solution of polyethylene glycol, fasted all night, and completed MGCE in the morning.

Healthy individuals (H group) were characterized by an absence of lesions in the stomach and small bowel through MGCE. Gastric inflammation (G group) was identified based on the Updated Sydney System (Dixon et al., 1996). Inflammation located in one out of three parts of the small bowel (duodenum, jejunum, and ileum) but not in the stomach was defined as “small intestinal inflammation” (I group).

We recorded (i) mucosal lesions, such as erosions; (ii) capillary lesions (angiodysplasias, petechiae); (iii) mucosal changes (erythema, edema, prominent mucosal folds); (iv) changes in villi (flat mucosa, coarsened villi); (v) lymphangiectasias/lymphocellular infiltrates.

### DNA Sampling

Samples of total DNA from fecal samples were extracted using a Fast DNA SPIN extraction kit (MP Biomedicals, Santa Ana, CA, United States) and stored at −20°C. The quality and quantity of isolated DNA were assessed by agarose gel electrophoresis and a NanoDrop^TM^ ND-1000 spectrophotometer (Thermo Fisher Scientific, Waltham, MA, United States).

### mNGS

DNA extracted from fecal samples was used for mNGS. Metagenomic libraries were constructed with a TruSeq^TM^ DNA Sample Preparation kit (Illumina, San Diego, CA, United States) and sequenced at Shanghai Personal Biotechnology (Shanghai, China) on an Illumina HiSeq system with a 150-bp paired-end protocol. Reads from each sample were retained and then merged by megahit (Li et al., 2015) and, if not, matched to human genome sequences (hg19) using Bowtie 2 (Langmead and Salzberg, 2012). The remaining results of merging were contigs of length ≥500 bp. Gene prediction was carried out based on MetaGeneMark (Noguchi et al., 2006) and then combined into a gene set. The protein sequences of genes were clustered to remove redundancy using cd-hit (Li and Godzik, 2006), with an identity cutoff of 90%, which resulted in unique gene sets. Reads from each sample were mapped to obtain their unique gene set. “Gene abundance” in each sample was the number of reads mapped to each gene sequence divided by the gene length. The percentage of gene abundance in the whole gene catalog was called the “relative abundance.” Diamond (Buchfink et al., 2015) was introduced to gene alignment and gene annotation in the National Center for Biotechnology Information-NR database with an e-value cutoff of 10-fold of the minimum value. Based on the alignment results, the algorithm of nearest common ancestors was taken into account for species annotation on genes. We annotated genes to species with in-house Perl scripts. The abundance of species in each sample was defined as the sum of gene abundance annotated to the same species. Functional classification was carried out by mapping to the Kyoto Encyclopedia of Genes and Genomes (KEGG) protein database and Clusters of Orthologous Groups of Proteins (COG) database (Tatusov et al., 2000) using KEGG Orthology-Based Annotation System (KOBAS) (Xie et al., 2011) and Diamond, respectively. Kruskal–Wallis analysis (Segata et al., 2011) was applied for classification and analyses of differentially expressed genes among dissimilar groups (*p* < 0.05, fdr < 0.05). In addition, principal component analysis (PCA) was used to analyze and visualize the sample distribution and discrimination among different disease groups.

### Differential Function of Expressed Genes

We compared any two sets of samples from H, G, and I groups by the relative abundance of genes. In each comparison, we removed the genes detected in less than five samples and continued analysis of abundance for the remaining genes using the Wilcoxon rank sum test, and the genes with test significance *p* < 0.05 were selected (Supplementary Table S1).

For additional analysis of differential function, the genes annotated in KEGG and COG databases were used for functional categories. For example, we used the differential genes of the G and H groups for analysis of hypergeometric distribution with unique genes as the background. Also, function annotations with *p* < 0.05 and fdr < 0.05 were enriched functions. Conversely, we counted the number of genes annotated in KEGG and COG functional categories and compared such functional categories between any two groups of samples by the Wilcoxon rank sum test. A function with *p* < 0.05 and fdr < 0.05 was determined to be a significantly differential function (Supplementary Table S2).

### Analysis of Dominant Microbiota and Networks in Bowel Inflammation and CG

Previously, we proposed an advanced computational framework (i.e., iENA) to provide a powerful network-analysis tool to quantify disease progression in an individual patient. Recently, we implemented an adjusted iENA using samples from healthy individuals as a network reference due to a limited number of individual samples and applied it in a proof-of-concept study on microbiota dynamics (Yu et al., 2019). Here, we used our approach to analyze the dominant microbiota and network to quantify different disease states using metagenomics data in three steps.

#### Constructing a Microbiota Edge-Network by iENA

After selecting reference samples, we constructed a co-expression network for one sample with our single-sample measurement of the Pearson correlation coefficient (sPCC) (Yu et al., 2017). Because of the absence of a background network for microbial communities, the top-ranked edges (i.e., one pair of species/genus) with strong relationships were selected as the background “nodes” for constructing the subsequent edge-network, which could consist of a conventional node-network or microbiota community (Wang et al., 2016; Sung et al., 2017). Furthermore, we continued quantification of the fourth-order correlation coefficient for each edge-pair (i.e., two species/genus pairs) by sPCC (Yu et al., 2017) for each single sample. Similarly, we only computed the correlations between the pre-selected high-ranked relations (edges) so that we could reduce unnecessary computations drastically. Finally, we obtained the microbiota-pair community/network corresponding to each sample from different disease states.

#### Recognizing and Quantifying Individual-Specific Microbiota Biomarkers

We selected top-ranked edge-pairs as edge-biomarkers, which have strong high-order compositional correlations and can be viewed as feature candidates represented as a set called “Markers.” In theory, each individual-specific biomarker is related to the clinical phenotype to some extent because the closely contacted candidates are identified in a certain disease state. As a warning signal (Chen et al., 2012; Zeng et al., 2013), we extended the dynamic network biomarker model in a manner of a single sample with its quantification criterion [i.e., composite index (sCI)] to quantify the disease state of each sample:

$$\text{sCI}=\bar{\frac{\sum_{x,y\in \text{Marker}}\left|\text{sPCC}\left(x,y\right)\right|}{\bar{\sum_{x\in \text{Marker},y\notin \text{Marker}}\left|\text{sPCC}\left(x,y\right)\right|}}}\times \bar{\sum_{x\in \text{Marker}}\left|x-u_{x}\right|}$$

where $\bar{{\text{PCC}}_{\text{in}}}$ is the average absolute value of PCC of a species/genus in the Marker group in one sample; $\bar{{\text{PCC}}_{\text{out}}}$ is the average absolute value of PCC of a species/genus between the Marker group and the other in one sample; $\bar{{\text{SD}}_{\text{in}}}$ is the average standard deviation in the Marker group. “Marker” was the set of dominant species/genus identified by iENA. Then, the sCI of individual markers could be used to indicate the possible disease signals when its value was sufficiently large.

#### Comparing Disease-Specific Markers and Their Discrimination for Disease

We could obtain the different individual-specific biomarkers to indicate disease-specific signals. Microbiota features that always present in the same disease group are more robust and representative in terms of disease specificity (Supplementary Table S3). Thus, we used the union set of microbiota features from patients with CG and small-bowel inflammation to characterize the varying microbiota community corresponding to disease states. In expectation of disease discrimination, we re-obtained the sCI value for each subject in different disease groups using such union markers, which indicated different etiologic mechanisms.

## Results and Discussion

### Study Populations

**FIGURE 1:**
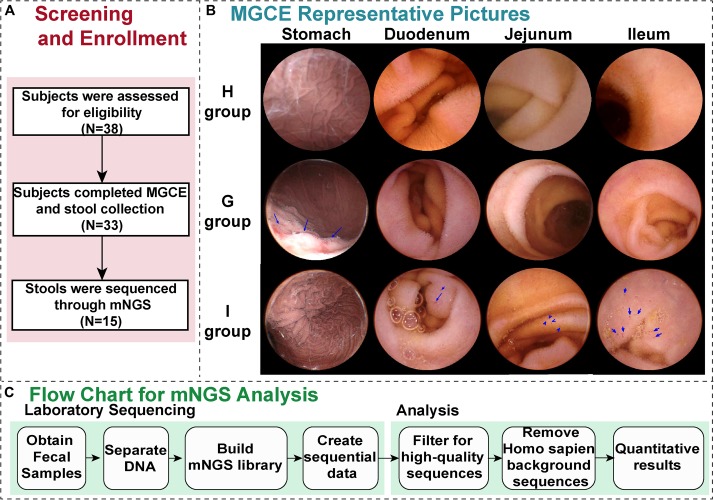
Generalization of the study. **(A)** The screening and enrollment of patients through the study. **(B)** Representative magnetically guided capsule endoscopy (MGCE) images (stomach, duodenum, jejunum, and ileum) of the H group (health group), G group (chronic gastritis), and I group (small bowel inflammation). **(C)** The protocol for the metagenomic next-generation sequencing (mNGS) assay. After samples of stool are received in the hospital, DNA is isolated, followed by construction of a metagenomic NGS library and sequencing.

From May 1, 2017 to September 1, 2018, 38 people with symptoms such as chronic abdominal pain, abdominal distention, and diarrhea participated in this study. They were screened across the Department of Gastroenterology of Shanghai Sixth People’s Hospital (Figure 1A).

Among them, 33 patients completed MGCE and stool collection, and the feces of 15 patients were processed through mNGS (Figure 1). The cohort primarily comprised, as defined by MGCE, individuals with gastric inflammation (G group), small bowel inflammation (I group), and a healthy population (H group), with five people in each group (Figure 1B and Table 1). The median age of the 15 patients was 53.0 years (Table 1). The proportion of male in these individuals was 40.0%, which was significantly high in patients with I group (26.7%) compared to H group (Supplementary Table S4). Recent studies observed no evidence for associations between gastrointestinal inflammation and gender (Khalili et al., 2020). The median value of alanine aminotransferase and total bile acid was 18 U/L and 3.35 μmol/L, respectively. Fourteen patients did have negative fecal occult blood in their feces (Table 1).

**TABLE 1:** Demographic and clinical characteristics of the 15 subjects.

Characteristic	Value
Age, years [median (range)]	53 (24–65)
Gender male/female	6/9
Diagnosis	
Health	5 (33.3%)
Gastritis alone	5 (33.3%)
Small intestinal inflammation	
Duodenum erosion lesion	1 (6.7%)
Jejunum erosion lesion	1 (6.7%)
Ileum erosion lesion	3 (20.0%)
Alanine aminotransferase	
Median (range), U/L	18 (4–26)
Total bile acid	
Median (range), μmol/L	3.35 (0.7–7.7)
Fecal occult blood testing	
Weakly positive	1 (6.7%)
Negative	14 (93.3%)

*Data are n (%) unless otherwise indicated.*

### Analyses of Microbiota Composition to Distinguish Between Small-Bowel Inflammation and CG

**FIGURE 2:**
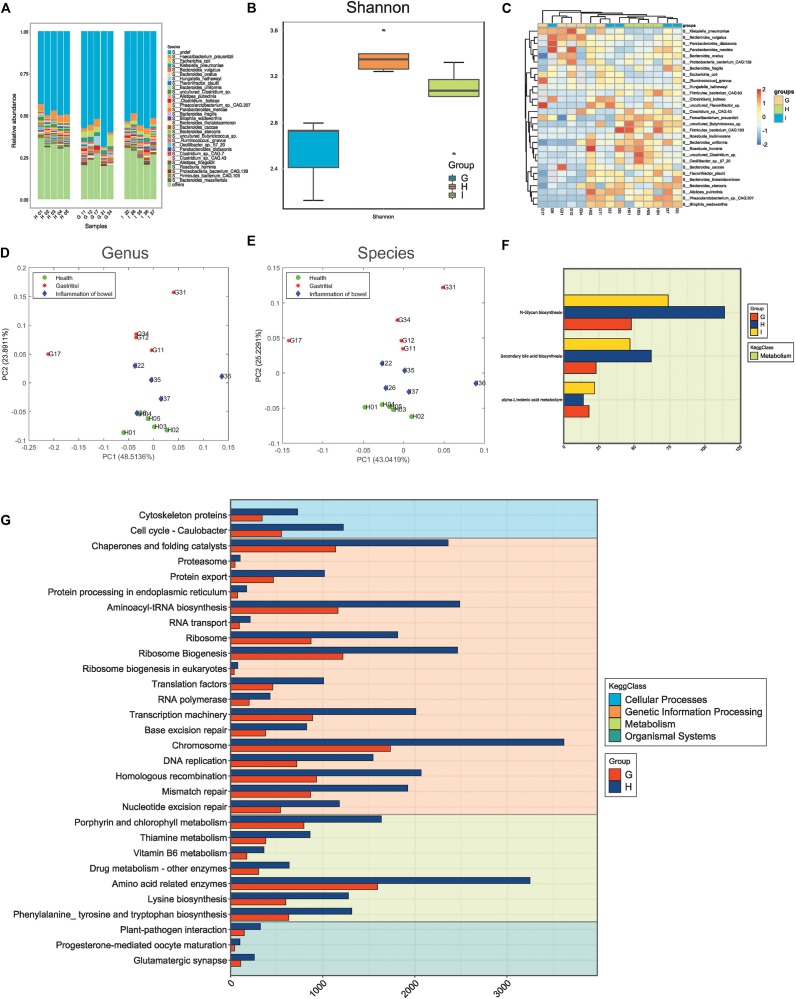
Microbiota composition distinguishing small-bowel inflammation and chronic gastritis. **(A)** The distribution of microbes in different samples. **(B)** The alpha-diversity of samples in different groups. **(C)** The abundance heatmap of differential microbes. **(D)** Principal component analysis (PCA) of samples with genus features. **(E)** PCA of samples with species features. **(F)** The differential functions between groups G (chronic gastritis), I (small bowel inflammation), and H (health group). **(G)** The differential functions between groups G and H.

Using mNGS, we demonstrated the quality of sequencing and obtained the microbe abundance through a standard pipeline. There were 52,820 differential genes between the G group and H group; 22,537 genes between the I group and H group; 17,670 genes between the G group and I group. We obtained two important results (Figure 2).

First, the distribution of different species in samples indicated the varying microbiota compositions. Also, the samples in the same group/state tended to have similar microbiota compositions (Figure 2A). The different species diversities displayed consistent measurements, and the microbiota compositions in healthy states were more diverse than those in the disease state (Figure 2B and Supplementary Figure S1), thereby suggesting remarkable disruption of the microbiota community when diseases occurred. The microbiota compositions in small-bowel inflammation were more diverse than those in CG (Figure 2B), which suggested that CG might be more serious than small-bowel inflammation at the microbiota level. A few microbes with different abundance between different groups were detected, although they seemed to not have effective discrimination together (Figure 2C).

Second, PCA supported the observation mentioned above that the microbiota community in healthy samples was consistent and similar, but had varying degrees of alterations in small-bowel inflammation or CG samples at the level of genus (Figure 2D) or species (Figure 2E). Functional analysis using metagenomics revealed that, compared with samples from healthy people, samples from people with small-bowel inflammation had dysfunction of the microbiome on metabolism (Figure 2F) (e.g., *N*-Glycan biosynthesis, biosynthesis of secondary bile acids, and metabolism of -Linolenic acid). Recent study has revealed that patients with IBD have a higher abundance of large-size glycans, as well as lower levels of galactosylation and fucosylation (Clerc et al., 2018). Those data are contrary to studies showing that levels of core fucosylation are increased in T cells in patients with IBD with an inflamed mucosa and in mice with colitis (Fujii et al., 2016). Moreover, supplements of related metabolites (e.g., diets of sage oil or hydrolyzed protein) can be efficacious treatment of chronic enteropathy. Rats with IBD or colitis have much lower mRNA levels of pro-inflammatory factors in the colon, resulting in a lower inflammatory response, significantly less colonic damage, and enhanced histological repair after administration of sage-oil diets (Reifen et al., 2015). Secondary bile acids consist of lithocholic acid and deoxycholic acid. A diet of hydrolyzed protein increases can lead to growth inhibition of *Escherichia coli and Clostridium perfringens* in rats suffering from chronic enteropathy. Moreover, the bile-acid producers *C. hiranonis* and *C. scindens* are related to diet-induced remission in rats with dextran sulfate sodium-induced colitis and children with IBD (Wang et al., 2019). CG samples showed greater changes in diverse KEGG functions (e.g., skeletal proteins in cellular processes, amino acid-related enzymes in metabolism, and glutamatergic synapses in organismal systems) (Figure 2G). The chromosome 1-related dominant trait is linked to resistance in mice to the autoimmune gastritis (Fujii et al., 2014). Besides, high levels of aneuploidy in chromosomes 4, 8, 20, and 17 (p53) have been detected in gastritis, dysplasia, intestinal metaplasia, and cancer samples (Williams et al., 2005).

### Analyses of Microbiota Communities to Distinguish Small-Bowel Inflammation and CG Using iENA

**FIGURE 3:**
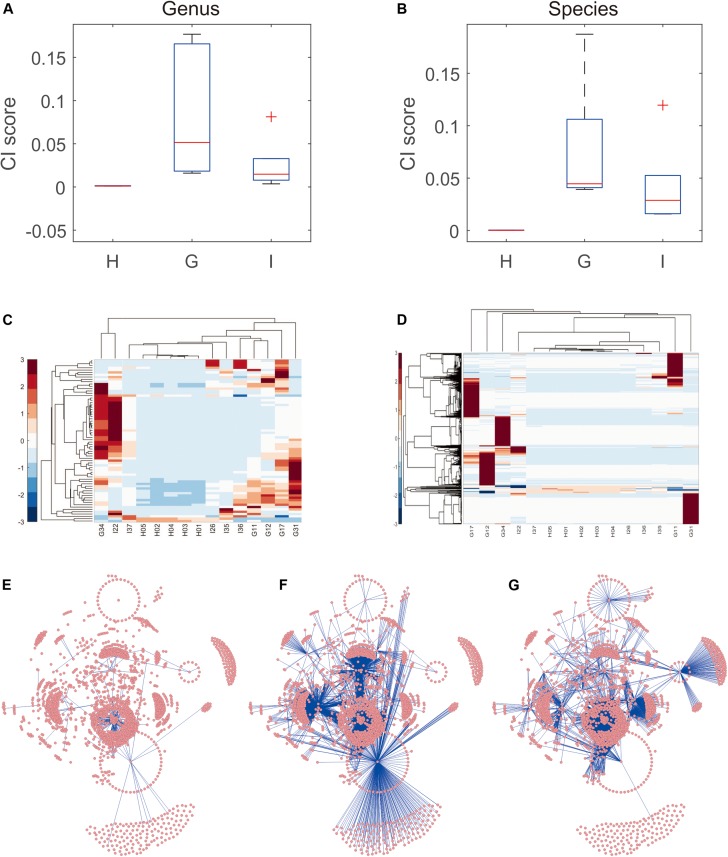
Microbiota community distinguishing small-bowel inflammation and chronic gastritis. **(A)** The distribution of CI scores distinguishes different groups on genus level. **(B)** The distribution of CI scores distinguishes different groups on species level. **(C)** The abundance heatmap of microbes in iENA markers. **(D)** The sPCC heatmap of microbe-pairs involved in iENA markers. **(E)** The microbe association network characterizing healthy state. **(F)** The microbe association network characterizing chronic gastritis state. **(G)** The microbe association network characterizing small bowel-inflammation state.

The data shown above indicated that the microbe abundance reflected the distribution of groups of samples (even though a simple combination of microbes could not be used to distinguish between disease states). Thus, the variance in microbe abundance was expected to supply more discriminative information, which could be detected by our proposed iENA approaches. Indeed, using one CI score with iENA-identified marker microbes, each sample could be assigned to a suitable group correctly. In accordance with the analysis stated above, CG samples had the highest scores, indicating the greatest variation/alteration in the microbiota community, whereas samples from healthy people had the lowest scores (which represented the stable microbiota community in individuals) (Figure 3A). More importantly, as reported in our previous study (Yu et al., 2019), the abundance of microbes could not be used to group samples with different phenotypes directly (Figure 3B). However, the co-expressed abundances of two microbes (e.g., edge makers detected using iENA) had the power to distinguish samples within dissimilar groups (Figures 3C,D). Such variation of the microbiota community can be used to quantify rewiring of microbiota networks. Hence, we reconstructed the correlation network of microbes corresponding to healthy samples, CG samples, and samples of small-bowel inflammation, respectively. In particular, the network-hub microbes in the different networks were the key microbiota involved in pathogenic processes (Supplementary Table S5). For example, CG-specific network-hub microbes tended to interact with many other microbes in the CG state but not in healthy or small-bowel-inflammation states (Figures 3E–G). Similarly, the small bowel inflammation-specific network-hub microbes tended to interact with many other microbes in the small-bowel-inflammation state, but not in the healthy or CG states (Figures 3E–G).

### Key Microbiota and Networks Revealed the Specific Pathogenesis Underlying Small-Bowel Inflammation and CG

Three important trends were observed in this part of our study.

First, for the key microbiota with differential abundance identified by conventional analysis, some well-known pathogenic microbes were discovered.

Microbes from the genera *Roseburia* and *Enterobacter* were related to *Helicobacter pylori* (HP) in gastritis and *Clostridioides difficile* infection (CDI) in IBD. The absence of *Roseburia* has been observed in HP-positive samples (Zhao et al., 2019), whereas the genus *Enterobacter* is dominant in HP-free patients (Hsieh et al., 2018).

Microbes from the genera *Blautia*, *Roseburia*, and *Flavonifractor* assist in the clinical classification and prognosis assessment of intestinal diseases, including IBS, IBD, and colorectal cancer (CRC) (Labus et al., 2017; Machiels et al., 2017; Ai et al., 2019; Gupta et al., 2019). The genera *Blautia* and *Flavonifractor* contribute to discrimination of IBS or CRC from controls (Labus et al., 2017; Ai et al., 2019). The decreased abundance of *Roseburia* and *Blautia* in feces specimens of patients with ulcerative colitis (UC) indicates a higher risk of pouchitis after ileal–anal pull-through surgery (Machiels et al., 2017). In particular, patients with IBD or CDI have lower levels of *Blautia* than those without CDI (Sokol et al., 2018). Also, the genus *Blautia* is enriched after 26 weeks of quadruple treatment with bismuth in patients with asymptomatic HP-related gastritis (He et al., 2019). Also, the abundance of *Roseburia* increases significantly after 1-week administration of vitamin D in Crohn’s disease (CD) cases (Schäffler et al., 2018).

In addition, *E. cloacae* is associated significantly with CD patients without antibodies to *Saccharomyces cerevisiae* (Kansal et al., 2019). Also, many microbes are associated with the immune response and therapeutic results in gut inflammation. For example, oral administration of *Citrobacter koseri* JCM1658 aggravates systemic allergic reactions and reduced numbers of intestinal T-helper-17 cells (Matsui et al., 2019).

Second, for the key microbiota with differential abundance identified by iENA, some were indeed candidate pathogenic microbes, though they did not have significantly different abundances.

Microbes from the family *Erysipelotrichaceae* and genus *Klebsiella* are strongly related to inflammation in the stomach and intestine. The family *Erysipelotrichaceae* is overgrown in mice with basal colitis (Chen et al., 2017) or HP infection (Pan et al., 2016). Conversely, the genus *Klebsiella* has not only been identified in case reports of acute phlegmonous gastritis (Kim et al., 2011; Matsuura et al., 2018) but is also the most familiar strain isolated from the small intestine of patients with small-intestinal bacterial overgrowth or UC (Pyleris et al., 2012; Tanaka et al., 2019). In particular, *K. pneumoniae* has been detected in the vast majority of patients with autoimmune gastritis (Furuta et al., 2018). Also, *K. oxytoca* is regarded as a pathogen that induces colitis (Högenauer et al., 2006), and its levels are increased in patients with active IBD compared with those in controls (Sánchez et al., 2013).

Third, for the key microbiota with intensive interactions with other microbes recognized by network-hub ranking, there is also considerable evidence of their pathogenic roles in gastroenteric inflammation or tumor.

Microbes are associated with inflammation in the gut, including *E. coli*-induced infectious ileitis (Alvarado et al., 2019) and the effect of *K. oxytoca* on gastritis and colitis, as mentioned above. *Acinetobacter lwoffii* has been isolated in a gastric-tissue culture in a case of acute phlegmonous gastritis (Kim et al., 2011). Non-HP organisms can induce gastritis, particularly inflammation or *lwoffi* (Rathinavelu et al., 2003) and *Listeria monocytogenes* in mice (Park et al., 2004).

Besides, the genera *Bacteroides* and *Bacillus* are strongly associated with patients with CG accompanied with a yellow tongue coating (Ye et al., 2016) or Barrett’s esophagus (Gutiérrez-Escobar et al., 2014). Moreover, different dietary strategies can alter the *Bacteroides* composition post-intervention in patients with superficial CG, such as a high-fat diet with increased *Bacteroides* (Wan et al., 2019) and wheat peptides/fucoidan with increased *B. intestinalis* (Kan et al., 2019). Some microbes are used in the treatment of diarrhea-predominant IBS and IBD, including *B. coagulans* MTCC 5856 (Majeed et al., 2016; Shinde et al., 2019) and *E. coli* Nissle 1917 (Sassone-Corsi et al., 2016).

In particular, microbe co-occurrence can have a combined effect during disease. The genera *Acinetobacter* and *Bacteroides* are increased significantly in IBD patients during the active phase (Andoh et al., 2012; Tang et al., 2015), though a massive amount of *Dialister invisus* has been identified in the state of inactivity or dormancy in the gut of patients with IBD (Schirmer et al., 2018). Also, the genus *Tyzzerella* and a greater abundance of *Dorea* and *Bacteroides* have shown good performance in distinguishing between patients with gastric cancer (Thomas et al., 2016; Qi et al., 2019) and rectal cancer (Dong et al., 2019). Also, *B. fragilis* has been identified to contribute to CRC development (Kwong et al., 2018).

## Conclusion

Recent studies have shown a high prevalence of small-bowel diseases (66% in Korea and 65.6% in China) in MGCE-examined subjects according to the data from multiple medical centers (Lim et al., 2015; Ding et al., 2019). Thus, increasing numbers of studies have focused on multiple microbiome compositions and their clinical applications through NGS, including CG, IBD, and IBS (Coker et al., 2017; Sokol et al., 2017; Vich Vila et al., 2018). However, the distribution of the fecal microbiome for explaining non-specific, mild inflammation (e.g., erosions) is largely unclear. We revealed the distribution of different species compositions, important alterations in microbiota, and metabolic dysfunction of the microbiome in fecal mNGS samples from patients with bowel inflammation or CG using a standard procedure. The metabolic dysfunctions in intestinal inflammation have been confirmed by other studies. Indeed, supplements of related metabolites can result in efficacious treatment of IBD, including diets of sage oil (rich in α-linolenic acid) and hydrolyzed protein (increased levels of secondary bile acids) (Reifen et al., 2015; Wang et al., 2019).

We used our own iENA approaches and discovered that two network-hub microbes with co-expression abundances were in ability to recognize samples in multiple clinical groups. In particular, the pivotal microorganisms in different networks were the key microbiota involved in pathogenesis. Recent reports have shown the pathogenic roles of microbes in bowel inflammation or CG, such as the genera *Acinetobacter* and *Bacteroides* in patients with IBD (Andoh et al., 2012; Tang et al., 2015).

The network-hub microbiota, significant rewiring of microbiota networks, and differential network analysis demonstrated that high-ranked iENA microbiota markers were disease specific and had particular pathogenic functions. Using mNGS combined with iENA represents a potential non-invasive step in the discrimination between gastrointestinal inflammation and healthy individuals. This approach could guide more targeted therapies against pathogens, assist in identification of disease phenotypes, and accelerate prognosis assessment of intestinal inflammation. Nevertheless, the pathogenesis, preferred timing of treatment, and patient population for clinical mNGS testing must be elucidated fully through further research.

## Data Availability Statement

The raw data generated for this study can be found in The National Omics Data Encyclopedia (NODE), project ID OEP000779.

## Ethics Statement

The studies involving human participants were reviewed and approved by the Ethics Committee of Shanghai Jiao Tong University Affiliated Sixth People’s Hospital, Shanghai, China. The patients/participants provided their written informed consent to participate in this study. Written informed consent was obtained from the individual(s) for the publication of any potentially identifiable images or data included in this manuscript.

## Author Contributions

J-SZ, JZ, and X-TY supervised the study. X-TY, X-YC, and JZ designed the experiments and analyzed the data. X-YC, H-NF, and H-WQ carried out the experiments. X-TY, X-YC, H-KZ, and LS undertook the data analysis. X-YC and X-TY prepared the manuscript. X-TY and X-YC revised the manuscript.

## Conflict of Interest

The authors declare that the research was conducted in the absence of any commercial or financial relationships that could be construed as a potential conflict of interest.

## Supplementary Material

FIGURE S1 Different diversities of samples.

TABLE S1 List of differential microbes.

TABLE S2 List of differential functions.

TABLE S3 List of markers from iENA.

TABLE S4 Baseline characteristics and distribution of participants.

TABLE S5 List of network-hub microbes.
