# Supplementary figures and images for: Rewiring of Microbiota Networks in Erosive Inflammation of the Stomach and Small Bowel
Source: Front Bioeng Biotechnol. 2020 May 13;8:299. doi: 10.3389/fbioe.2020.00299 (PMC7237573; doi:10.3389/fbioe.2020.00299)

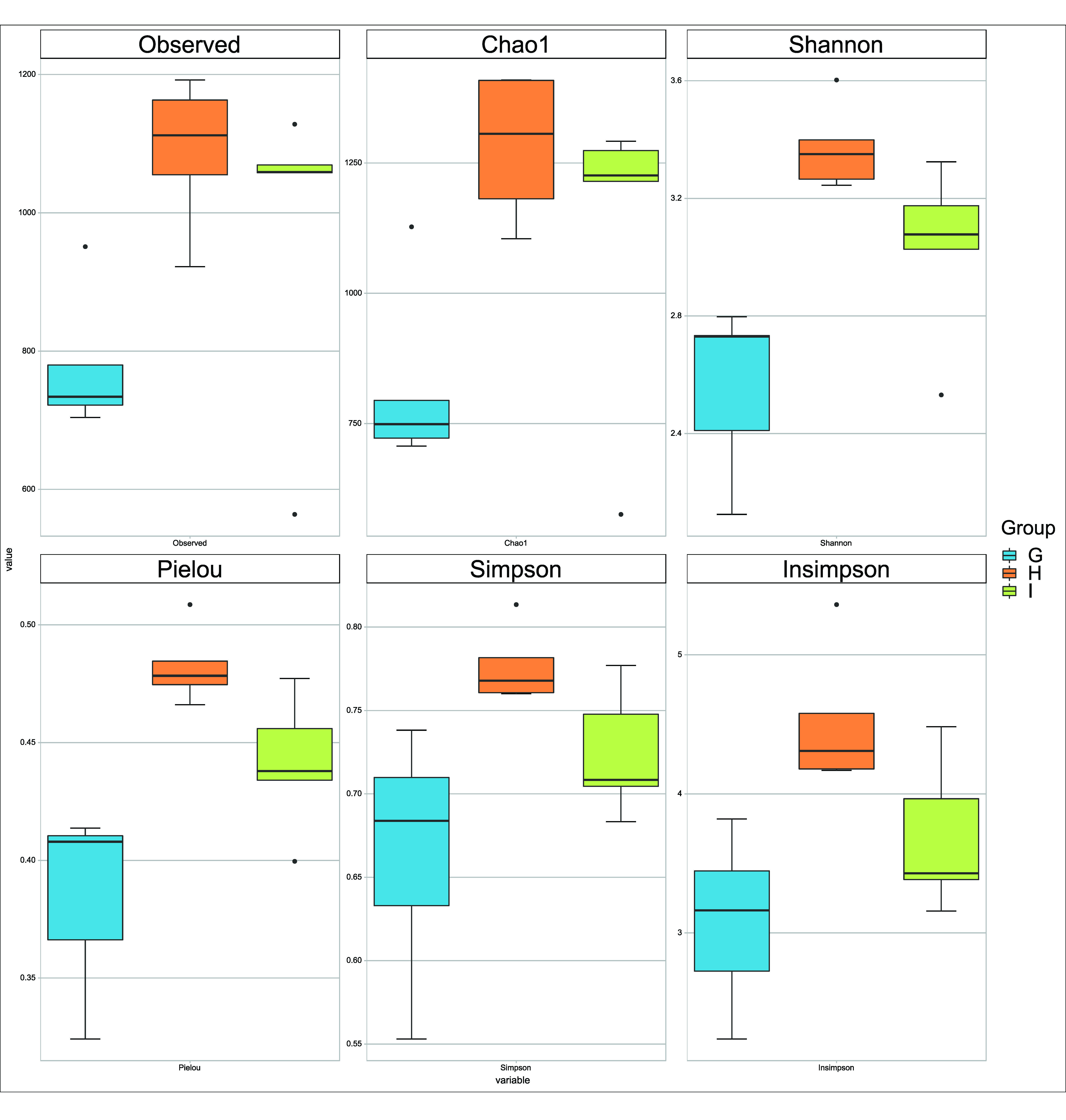

Supplement: FIGURE S1 — Different diversities of samples. [file Image_1.JPEG]
